# A Case of Collapsed Stent Graft, Severe Lower Limb Ischemia, and Ruptured Abdominal Aortic Aneurysm Due to Type B Acute Aortic Dissection 3 Years after Endovascular Aneurysm Repair

**DOI:** 10.3400/avd.cr.19-00142

**Published:** 2020-09-25

**Authors:** Yusuke Motoji, Takayoshi Kato, Jun Seki, Kosuke Tsumura, Shinji Tomita, Yasuhide Okawa

**Affiliations:** 1 Department of Cardiovascular Surgery, Gifu Heart Center

**Keywords:** endovascular aneurysm repair, acute aortic dissection, severe lower limb ischemia, abdominal aortic aneurysm rupture

## Abstract

We report a case of stent graft occlusion, severe lower extremity ischemia, and ruptured abdominal aortic aneurysm due to type B acute aortic dissection 3 years after endovascular aneurysm repair. He admitted our hospital because of abrupt back pain and dysesthesia of bilateral lower limb. Contrast-enhanced computed tomography (CT) scan showed type B acute aortic dissection and occlusion of the stent graft due to dynamic compression by the false lumen. Emergent right axillo-bifemoral bypass operation was done for his critical limb ischemia. Immediately after the successful operation, he fell into shock vital and dissecting abdominal aortic aneurysm rupture was revealed by CT scan. We performed the stump occlusion of the infrarenal abdominal aorta and the bilateral common iliac arteries by abdominal midline incision. Postoperative myonephropathicmetabolic syndrome due to the left ischemia resulted in amputation of his left lower leg for lifesaving. While EVAR cases are increasing, various its complications come to be reported. We consider that this case report might be cautious about the indication of EVAR for the younger generation. (This is a translation of Jpn J Vasc Surg 2019; 28: 367–371.)

## Introduction

Vascular prostheses have been widely used in the treatment of aortic aneurysms. Recently, the number of patients receiving a stent graft (SG) for abdominal aortic aneurysm (AAA) has been increasing each year in Japan.^1)^ Various complications have been reported. The literature has reported six cases of collapsed SG due to Stanford type B acute aortic dissection (AAD(B)) after endovascular aortic repair (EVAR).^2–5)^ We report here a case of AAD(B) complicated by previously implanted endovascular graft collapse and aneurysm rupture. To the best of our knowledge, this is the first report of such a case.

## Case

**Figure 1:**
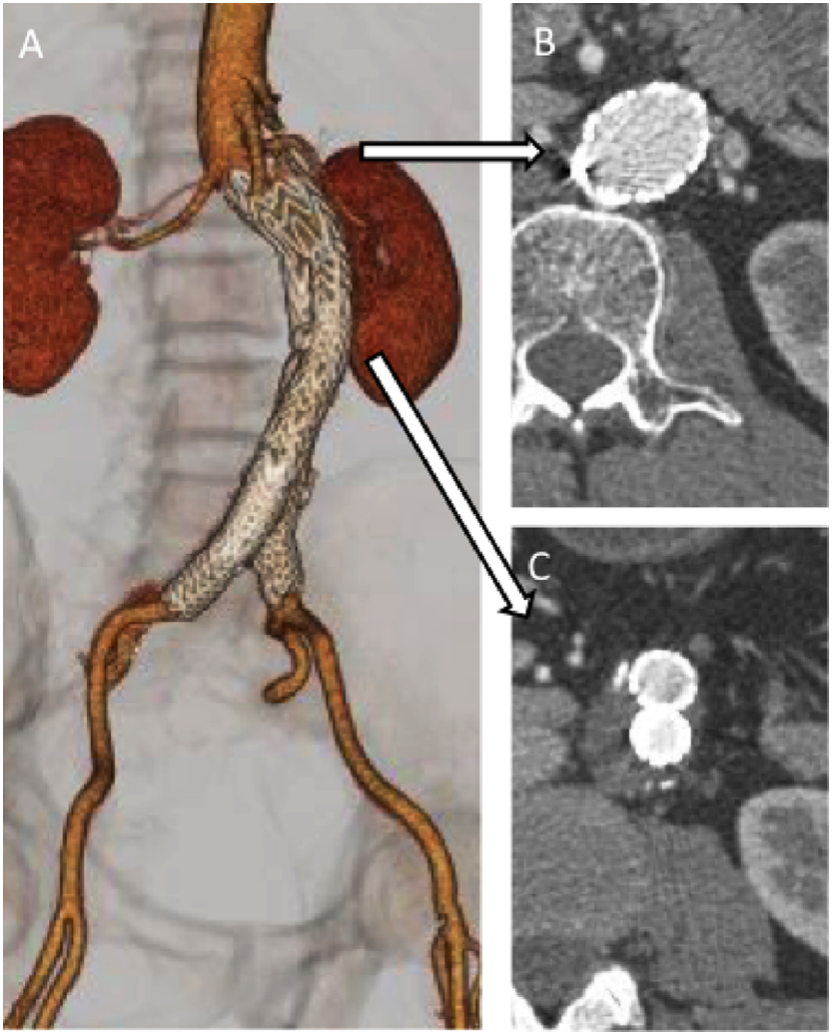
Fig. 1 (**A**) Aortic aneurysm has disappeared on CT scan taken before the onset of dissociation. (**B**), (**C**) The abdominal aortic aneurysm found before EVAR has almost disappeared.

**Figure 2:**
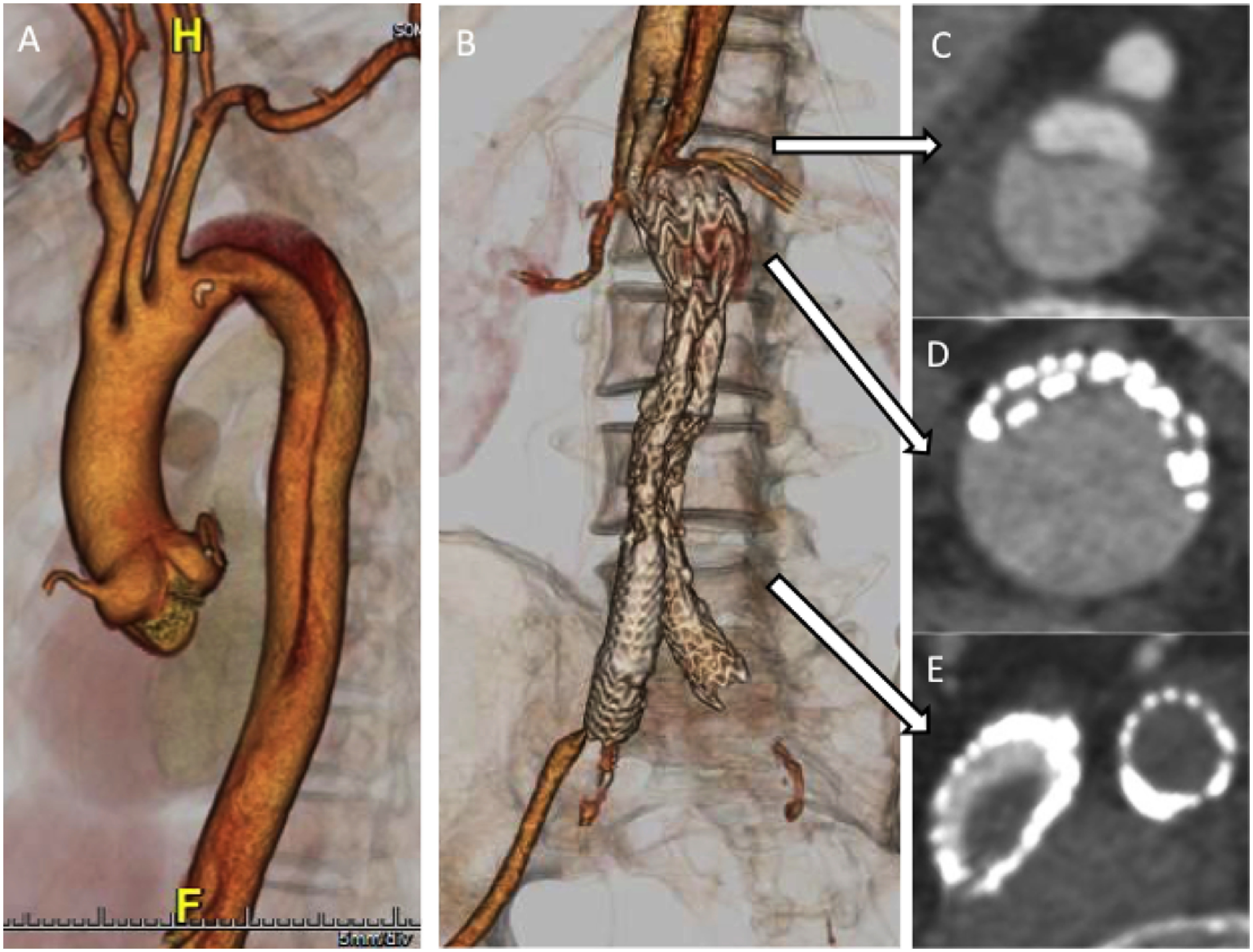
Fig. 2 (**A**), (**B**) At the onset of acute type B aortic dissection, CT scan showed the dissection extended from the descending aorta to the bilateral common iliac artery. Aortic dissection progressed retrogradely to the descending aorta. There was no obvious re-entry. The maximum short diameter of the aneurysm is 47 mm and the false lumen diameter is 37 mm, which imply a high value of the false lumen pressure. (**C**) The entry was observed near the superior mesenteric artery origin. (**D**) The stent graft was compressed and completely occluded by the false lumen pressure. (**E**) The blood flow to the left leg is completely interrupted.

A 62-year-old man was admitted to our hospital with acute back pain and paresthesia of the lower limbs. The patient had a history of EVAR using a Gore Excluder device (W. L. Gore & Associates, Flagstaff, AZ, USA) under a diagnosis of 48-mm infrarenal AAA with rapid expansion 3 years earlier, and the latest follow-up computed tomography (CT) showed that the treated aneurysm had almost disappeared (**Fig. 1**). He reported symptoms of ischemia in the leg, such as pain, pallor, paresthesia, paralysis, and pulselessness of the distal arteries. Emergent CT showed AAD(B) with a false lumen compressing the SG (**Fig. 2D**). The level of dissection was from the distal arch to the main body of the SG (**Fig. 2E**). CT showed that the major entry was located near the superior mesenteric artery (SMA) (**Fig. 2C**), with some minor re-entries in the descending aorta (**Fig. 2A**). Under the renal artery, a new dissecting AAA was seen with a maximum short diameter of 47 mm (false lumen diameter, 37 mm) (**Fig. 2B**).

### First operation

**Figure 3:**
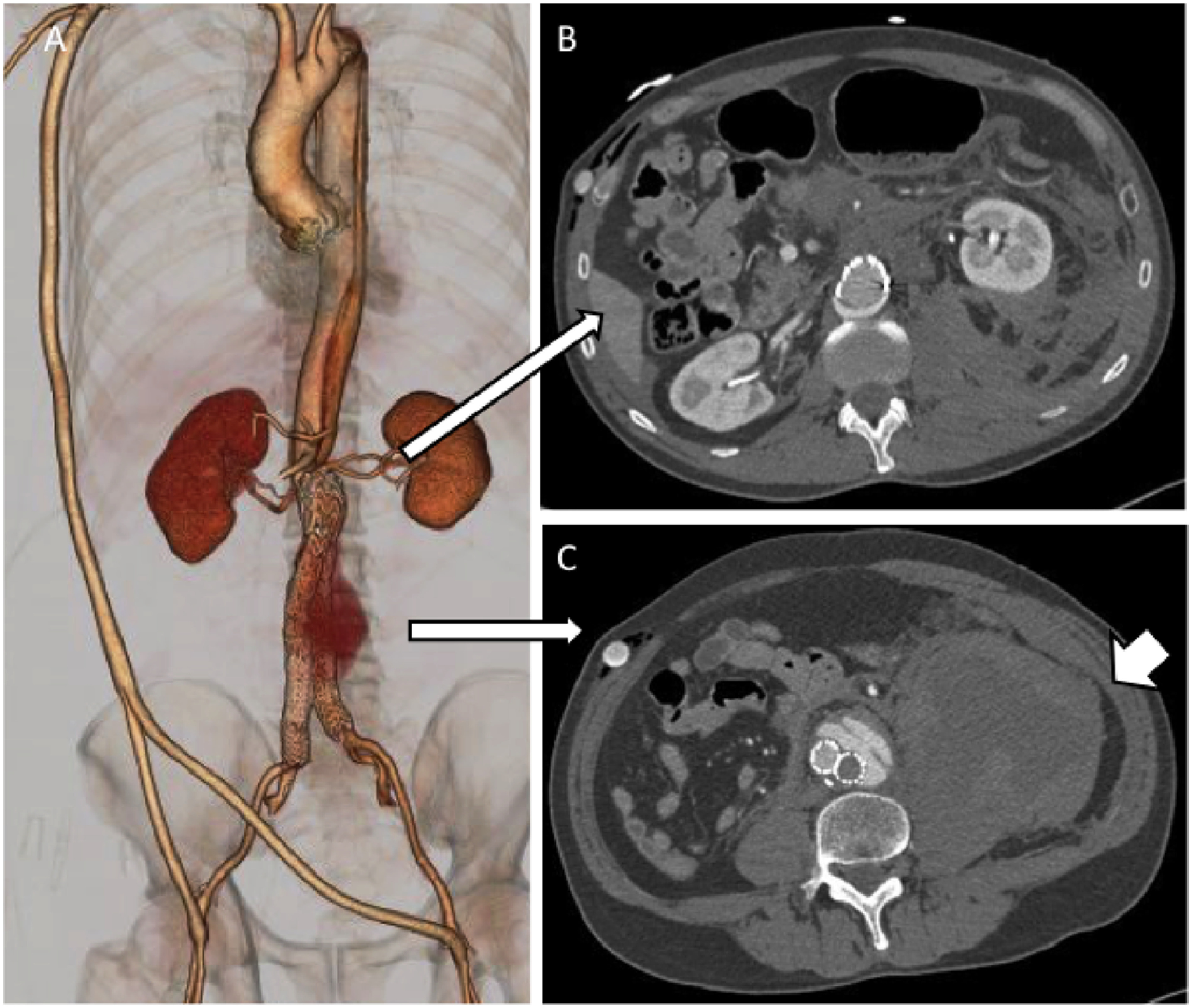
Fig. 3 (**A**), (**B**) Contrast-enhanced CT showed that the stent graft was re-expanded by the retrograde blood flow. (**C**) It was revealed that the dissecting abdominal aortic aneurysm has ruptured after the right axillo-bifemoral bypass operation. A large amount of hematoma was observed in the left retroperitoneum at the position of terminal aorta.

Under general anesthesia, right axillo-bilateral femoral artery bypass (AxBFB) was emergently performed using a 10×8-mm Gelsoft ERS Equi-Flo Bifurcated Axillo-Bifemoral graft (Gelsoft; Vascutek, Scotland, UK) and leg ischemia resolved. However, about 20 min after leg reperfusion, blood pressure suddenly decreased. We transfused 6 units of red blood cells, and hemodynamics improved. Conspicuous induration of the abdomen was also observed. CT for close-up examination showed a ruptured dissecting AAA (**Fig. 3**). We took the patient to the operation theater again.

### Second operation

A midline abdominal incision revealed massive intraperitoneal hemorrhage and retroperitoneal hematoma. The suprarenal abdominal aorta was clamped 8 min after the operation started. The intima seemed severely impaired, and the adventitia was tightly adherent to surrounding tissues. We considered that graft replacement would be too difficult, so we performed stump closure of the abdominal aorta and common iliac arteries.

### Postoperative course

Myonephropathic metabolic syndrome due to limb ischemia and reperfusion injury was noted, requiring intensive care. Fasciotomy was performed to the left lower limb because anterior tibial compartment pressure exceeded 50 mmHg. However, left lower limb amputation was performed 16 days after surgery due to the left lower limb. The patient’s general condition gradually recovered, and he was transferred to another hospital for rehabilitation with an artificial leg on the 92nd postoperative day. He is currently being followed up in our outpatient clinic. As of the time of writing this report, no aneurysm has been identified at the closed aortic stump and no enlargement in diameter of the descending aorta has been observed.

## Discussion

According to a 2013 annual report in Japan, 10,778 of 19,216 AAAs were treated with SG.^1)^ Thirteen years have passed since EVAR was introduced in Japan. Many complications due to EVAR have recently been reported. Various types of complication, such as endoleaks, arterial injury, embolism, stent stenosis/occlusion, and retrograde aortic dissection, have been observed. As for retrograde aortic dissection, Kpodonu et al.^6)^ observed severe complications in 7 (2.4%; mean observation period, 202 days) among 287 patients who underwent EVAR. In the literature, only six cases of collapsed SG due to AAD(B) after EVAR have been reported.^2–5)^ However, no cases showing rupture after revascularization have been reported.

Treatment options for the above conditions are broadly classified into endovascular treatment and surgical revascularization. van Keulen et al.^2)^ and Iyer et al.^3)^ chose endovascular treatment. They succeeded in passing a wire through the displaced stent and closed the entry to the thoracic aorta by adding a new SG. Compared with surgical revascularization, endovascular treatment could be completed in a short time and is minimally invasive. However, if the wire operation proves difficult, the patient risks an excessively long ischemic time. CT showed the major entry near the SMA, so we chose surgical revascularization instead of endovascular graft. On the other hand, several authors have reported surgical revascularization.^4,5)^ Re-expansion of the SG can reportedly be obtained by a retrograde increase in true lumen perfusion pressure.^4,5)^ AxBFB is a simple and reliable method of revascularization. However, its long-term patency rate and risk of non-infection are lower compared with those of anatomical revascularization. In this case, the SG was almost completely occluded, and severe limb ischemia was seen. We therefore selected AxBFB for earlier revascularization. We considered two factors that may have contributed to aneurysm rupture immediately after reperfusion. The first factor was the very high false lumen pressure, which invaded the abdominal aortic wall beyond the proximal landing zone. Increased false lumen pressure induced by retrograde flow due to type Ia or III endoleak is thought to be a cause of SG occlusion and subsequent rupture. The other factor is SG radial force. Re-expansion of the SG due to an unexpected increase in false lumen pressure might contribute to aneurysm rupture. For the above reasons, excessive pressure could have been applied to the aneurysm wall, leading to rupture.

Cambria et al.^7)^ reported that coexistence of atherosclerotic aneurysm and acute dissection appears to increase the risk of aortic rupture in both proximal and distal aortic segments. Even if the aneurysm disappears after EVAR, the three-layer structure of the aorta is already degenerated. If blood vessel undergoes dissection, aortic rupture seems likely to result. Year by year, the use of EVAR has been increasing, and early postoperative results of EVAR are better compared with those of surgical treatment. However, about 26% of patients need re-intervention,^8)^ and 0.8–5.9% of patients need open surgery^9)^ because of complications such as aneurysm enlargement and infection in the remote stage. Late open conversion often requires supraceliac or suprarenal aortic clamping. Kouvelos et al.^10)^ reported a 30-day mortality rate of 9.1% (elective surgery, 3.2%; emergency surgery, 29.2%). Causes of poor surgical outcome have been reported as a need for thoracoabdominal aortic replacement, risk of acute renal failure due to suprarenal aortic clamp, and inflammation around the proximal neck after EVAR.

In our case, anatomical graft replacement was difficult because of severe intimal damage and adhesion. Aortic stump closure carries a risk of enlargement and infection. However, we chose this procedure to keep the patient out of danger. If circulatory dynamics had been stable, anatomical graft replacement would have been preferable. Since no major re-entry was apparent, false cavity pressure was expected to be high. As a result, the diameter of the descending aorta might have been increased. Three years have passed since the operation, and the patient has been followed up on an outpatient basis. No major complications were encountered. However, careful observation will need to be continued.

Given that a wide variety of complications beyond endoleak and infection can occur, caution is required about the many complications of EVAR.

## Conclusion

We have presented a rare case of AAD(B) complicated by previously implanted endovascular graft collapse. Revascularization was performed using an axillo-bilateral femoral bypass. However, a drastic complication of the dissecting aneurysm rupture was observed. This manifestation might be considered as a severe late complication after EVAR.

## Additional Note

The patient provided permission for publication of the features of his case, and we have ensured that his identity is protected.

## Notes

This is a translation of Jpn J Vasc Surg 2019; 28: 367–371.
